# Epigenetic cues modulating the generation of cell‐type diversity in the cerebral cortex

**DOI:** 10.1111/jnc.14601

**Published:** 2018-11-22

**Authors:** Nicole Amberg, Susanne Laukoter, Simon Hippenmeyer

**Affiliations:** ^1^ Institute of Science and Technology Austria Klosterneuburg Austria

**Keywords:** cell‐type diversity, cerebral cortex, gliogenesis, lineage progression, neurogenesis, radial glial progenitor cells

## Abstract

The cerebral cortex is composed of a large variety of distinct cell‐types including projection neurons, interneurons, and glial cells which emerge from distinct neural stem cell lineages. The vast majority of cortical projection neurons and certain classes of glial cells are generated by radial glial progenitor cells in a highly orchestrated manner. Recent studies employing single cell analysis and clonal lineage tracing suggest that neural stem cell and radial glial progenitor lineage progression are regulated in a profound deterministic manner. In this review we focus on recent advances based mainly on correlative phenotypic data emerging from functional genetic studies in mice. We establish hypotheses to test in future research and outline a conceptual framework how epigenetic cues modulate the generation of cell‐type diversity during cortical development.

**Abbreviations used**
5caC: 5‐carboxylcytosine
5fC: 5‐formylcytosine
5hmC: 5‐hydroxymethylcytosine
5mC: 5‐methylcytosine
BAF: Brahma‐associated factor
bRG: basal radial glial progenitor
CBP: CREB binding protein
Cdkn1c: cyclin‐dependent kinase inhibitor 1c
CHD: chromodomain‐helicase‐DNA‐binding
cPcdh: clustered protocadherin
CP: cortical plate
CTCF: CCCCTC‐binding factor
Dlk1: delta‐like homologue 1
DNA: deoxyribonucleic acid
DNMT: DNA methyltransferase
EED: embryonic ectoderm development
EZH2: enhancer of zeste 2
HAT: histone acetyl transferase
HDAC: histone deacetylase
Igf2: insulin‐like growth factor 2
IP: intermediate progenitor
IZ: intermediate zone
lincRNA: long intergenic non‐coding RNA
lncRNA: long non‐coding RNA
MADM: Mosaic analysis with double markers
NESC: neuroepithelial stem cell
NF1: nuclear factor 1
NICD: Notch intracellular domain
Norad: non‐coding RNA activated by DNA damage
NSC: neural stem cell
NuRD: nucleosome remodeling deacetylase
OPC: oligodendrocyte progenitor cell
oRG: outer radial glia
PRC: polycomb repressive complex
PUM: Pumilio
RCoR: REST co‐repressor
RGP: radial glial progenitor
RNA: ribonucleic acid
SCPN: subcerebral projection neuron
SUZ12: suppressor of zeste 12
SVZ: subventricular zone
TAD: topologically‐associated domains
TDG: thymine DNA glycosylase
TET: ten‐eleven translocation protein
VZ: ventricular zone
Zac1: zinc finger protein regulating apoptosis and cell cycle arrest

The human cerebral cortex is the seat of our cognitive abilities and composed of an extraordinary number of neurons and glial cells. A remarkable heterogeneity in the cortical projection neuron types has been described (Lein *et al*. 2017; Luo *et al*. 2017; Zeng and Sanes 2017), yet the identity and development of the neuronal classes that constitute the cortical microcircuits appears to a large extent genetically hard‐wired (Lodato and Arlotta 2015). During development, the mammalian cerebral cortex derives from the embryonic neuroectoderm. At the end of neurulation and neural tube closure the neuroepithelium is composed of neuroepithelial stem cells (NESCs) from which all subsequent neural progenitor cells and their neuron lineages derive. NESCs initially amplify their pool in fast cell cycle divisions before they transform into radial glial progenitors (RGPs) (Taverna *et al*. 2014). RGPs have been demonstrated to be the main source in the developing cortex for the vast majority of cortical excitatory neurons, transient amplifying progenitors such as intermediate progenitors (IPs) (Kowalczyk *et al*. 2009; Vasistha *et al*. 2014), outer subventricular zone (SVZ) radial glial progenitors (oRGs aka basal RGs or bRGs) (Beattie and Hippenmeyer 2017), a subset of glial lineages and adult SVZ stem cells (Bayraktar *et al*. 2015). The apical processes of RGPs serve as a scaffold for nascent cortical neurons, which migrate from the ventricular zones (VZ)/SVZ through the intermediate zone, in order to reach the cortical plate (CP) (Evsyukova *et al*. 2013; Hippenmeyer 2014). Cortical layering occurs in an ‘inside‐out’ fashion whereby earlier born neurons populate deep layers and later born neurons progressively occupy upper layers (Angevine and Sidman 1961). Thus, the sequential generation of discrete cell fates, and concerted migration to correct laminae, is critical for the assembly of the neocortex.

## Radial glial progenitors generate cell‐type diversity in the cerebral cortex

**Figure 1:**
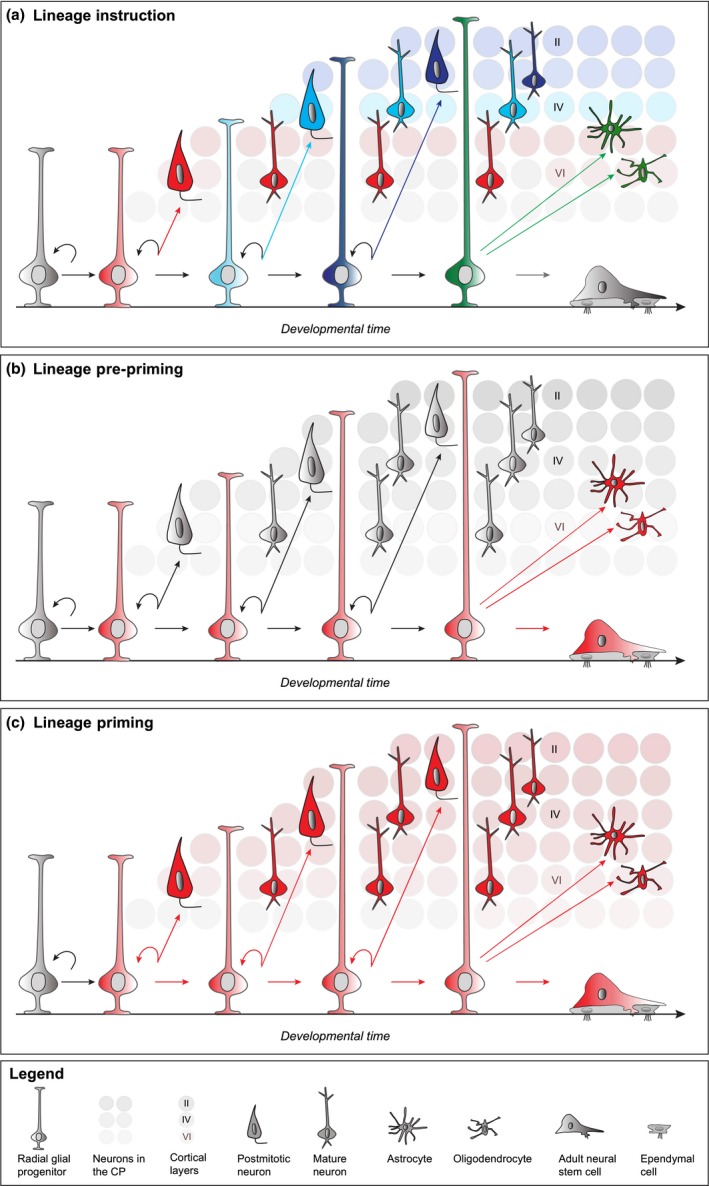
Epigenetic regulation of RGP‐mediated generation of cell‐type diversity. (a) Lineage instruction: epigenetic factors in RGPs together with local factors work in an orchestrated manner to instruct distinct neuronal/glial fates. These factors act only at a specific time window and during a distinct step of cell‐type generation. (b) Lineage pre‐priming: the entire RGP lineage is pre‐primed by a specific epigenetic factor in early RGPs throughout the course of development. However, the functional impact manifests only at a later stage of lineage progression (e.g. during glial differentiation or adult NSC proliferation). (c) Lineage priming: the entire lineage is instructed by the functional impact of an epigenetic factor that is uniformly present throughout all stages of development. In combination with local niche‐derived factors the epigenetic mark exerts its function on progenitor state, RGP proliferation and the entire successive lineage.

The concerted production of the correct number and diversity of neurons and glial cells is essential for intricate cortical circuit assembly and an exquisite balance between RGP proliferation/differentiation must be reached in order to generate a neocortex of appropriate size. To elucidate the precise patterns of RGP division, neuron, and glial cell production, Mosaic analysis with double markers (MADM)‐based quantitative clonal analysis has recently been performed (Zong *et al*. 2005; Hippenmeyer 2013; Gao *et al*. 2014). This systematic clonal analysis suggests that the behavior of RGPs is remarkably coherent and predictable across all developmental stages. RGPs in the neurogenic phase do not undergo terminal differentiation in a stochastic manner but rather follow a defined program of cell cycle exit resulting in a unitary output of about 8–9 neurons per individual RGP. The size of asymmetric neurogenic clones is however similar across neocortical areas with distinct functions, providing evidence that the unitary neuronal output is a general property of cortical RGPs. Upon completion of neurogenesis, a defined fraction of individual RGPs proceed to gliogenesis whereby about 1 in 6 neurogenic RGPs produce glia – astrocytes and/or oligodendrocytes – indicating a coupling between gliogenesis and neurogenesis at a predictable rate. While the MADM‐based lineage analysis revealed definitive quantitative ontogeny of neocortical excitatory neurons and glial cells (Gao *et al*. 2014), the cellular and molecular mechanisms dictating neural progenitor cell lineage progression are not well understood (Beattie and Hippenmeyer 2017). Major progress has been made in classifying cell‐types based on single cell transcriptome analysis (Lein *et al*. 2017; Luo *et al*. 2017; Zeng and Sanes 2017), however it remains elusive which neuronal and glial cell types arise from an individual progenitor cell. Furthermore, the regulatory modules and epigenetic cues that furnish RGPs with their precise programs to generate projection neuron and glial cell diversity are poorly defined. In this review we discuss recent progress advancing our conceptual understanding and stimulating new hypotheses that can be tested in future research. Epigenetic signaling cues include specific chemical modifications which modulate chromatin structure and organization. The major biochemical signaling pathways organizing the chromatin architecture include DNA methylation, histone modifications, or expression of long non‐coding RNAs (Di Croce and Helin 2013; Yao *et al*. 2016). Cells combine these features, defining the epigenetic code, in a cell‐type and temporally specific manner. The code determines whether the chromatin configuration at particular genomic loci exerts an active state, characterized by opening of chromatin allowing access by the transcriptional machinery; or repression, defined by chromatin condensation (Kouzarides 2007; Karlić *et al*. 2010; Portela and Esteller 2010). Progressive modification of the epigenetic landscape in RGPs and nascent cortical neurons controls transcriptional accessibility of specific target genes (Albert *et al*. 2017). Epigenetic cues may instruct neural stem cell lineage progression in a number of ways and below we outline three major hypothetical conceptual frameworks: (i) Lineage instruction – an epigenetic factor acts during a specific developmental window to initiate the differentiation into a specific cell‐type; (ii) Lineage pre‐priming – an epigenetic factor is present throughout development but is only instructive at later stages to direct the development of a certain cell‐type e.g. glial cells; (iii) Lineage priming – an epigenetic mark affects the development of an entire lineage and has functional impact on all cell‐types generated within this lineage (Fig. 1). Considering these hypothetical frameworks we focus on specific key questions in light of recent correlative phenotypic data obtained from genetic studies in mice: How do epigenetic regulatory cues modulate the *quantitative* and *qualitative* output of a single cortical stem cell? Which signaling pathways are transcriptionally regulated in order to modulate stem cell potential over time? And in a broader context, how do epigenetic factors regulate lineage priming and/or instruction in the course of RGP‐mediated generation of cortical cell‐type diversity?

## DNA methylation and hydroxymethylation during cortical projection neuron development

DNA methylation represents a critical epigenetic mark modifying DNA‐protein interactions and thus controlling transcriptional states and cellular identity. Methylation of cytosine (5‐methylcytosine, 5mC), at CpG dinucleotides modulates core epigenomic processes including gene expression, imprinting, X‐inactivation, silencing of repetitive elements and regulation of heterochromatin (Robertson and Wolffe 2000; Jaenisch and Bird 2003; Bergman and Cedar 2013). DNA methylation most often occurs at CpG dinucleotides, but also marks CpH (H being any other nucleotide than G), particularly in a CpA context (Lister *et al*. 2013). The methylation pattern during embryogenesis is highly dynamic and exhibits a remarkable degree of tissue and cell‐type specificity. Postmitotic neuron maturation requires accumulation of methylation marks at both, CpG and CpH sites. Lineage specification was shown to correlate with differences in CpA methylation patterns (Sharma *et al*. 2016).

In mammals, DNA methylation is catalyzed by a family of DNA methyltransferases (DNMTs), including maintenance methyltransferase DNMT1, *de novo* methyltransferases DNMT3a and 3b, and the catalytically inactive DNMT3L (Lyko 2018). *Dnmt1* is expressed throughout cortical development, with increasing expression from progenitor cells to neurons (Hutnick *et al*. 2009). *Dnmt1* controls both, quantitative and qualitative RGP neuron and glia output. *Dnmt1*–deficient RGPs display upregulation of genes involved in apoptosis and downregulation of genes required for neuronal differentiation and maturation. Consequently, *Dnmt1* mutant mice display cortical degeneration, defective neuronal layering, absence of barrel fields and precocious astrocyte generation (Fan *et al*. 2001, 2005; Golshani *et al*. 2005; Hutnick *et al*. 2009). The precise function of DNA methylation in RGP lineage control is currently not known but lineage instruction at the neurogenic to astrocytic transition was analyzed in more detail. During early neurogenesis, CpG sites within promoters of genes regulating gliogenesis, e.g. *Gfap*, are methylated in a *Dnmt1*‐dependent manner, thus preventing their expression (Takizawa *et al*. 2001). At progressively later neurogenic stages, Notch activation in RGPs induces the expression of nuclear factor I, which displaces DNMT1 at the *Gfap* promoter (Namihira *et al*. 2009). Therefore methylation of astrogenic gene promoters is selectively abolished (Takizawa *et al*. 2001), which in turn leads to binding of STAT1/3 heterodimers and the promotion of astrogenic gene expression (Fan *et al*. 2005; Hatada *et al*. 2008). In the oligodendrocyte lineage, *Dnmt1* regulates oligodendrocyte specification not only by silencing genes involved in oligodendrocyte precursor cell (OPC) proliferation and neuronal differentiation; but also by orchestrating alternative splicing events (Moyon *et al*. 2016). In mice, the ablation of *Dnmt1* results in severe hypomyelination. At the cellular level OPC maturation is impaired because of misfolded proteins and subsequent activation of endoplasmic reticulum stress response (Moyon *et al*. 2016). Furthermore, based on loss of function data *Dnmt1* appears to be required for (olfactory bulb‐destined) neuroblast generation from adult neural stem cells (NSCs) (Noguchi *et al*. 2016) albeit the precise mechanisms remain elusive and require further studies.

The function of *Dnmt3a/b* in corticogenesis is even less clear. The expression patterns of *Dnmt3a* and *Dnmt3b* are distinct from each other, indicating non‐redundant functions in cortical neurogenesis. *Dnmt3b* is highly expressed in early progenitors from E10.5 until E13.5 and in differentiated neurons from E17.5 onwards. *Dnmt3a* is robustly expressed in immature neurons from E13.5 until E17.5 but shows low expression in mature neurons (Feng *et al*. 2005; Watanabe *et al*. 2006). Recent studies provide evidence for *Dnmt3b*‐dependent methylation of the promoters of clustered protocadherin (*cPcdh*) isoforms, a family of adhesion molecules (Chen and Maniatis 2013), at early stages of neurogenesis. Strikingly, the loss of *Dnmt3b* results in altered expression of *cPcdh* isoforms (Toyoda *et al*. 2014). These findings are intriguing in the context of the possible functional role of cell lineage in modulating the preferential connectivity of clonally related cortical projection neurons (Yu *et al*. 2009; Li *et al*. 2012). Indeed, a transient increase of reciprocal connections of clonally related neurons in the somatosensory barrel cortex depends on functional *Dnmt3b* regulating proper *cPcdh* isoform expression (Tarusawa *et al*. 2016). To which extent *Dnmt3b* activity is required in proliferating RGPs to prime the lineage and thus clonally related progeny remains a key question for future studies.

Hydroxymethylation and in particular 5‐hydroxymethylcytosine (5hmC), which is preferentially detected in intragenic regions, is an abundant epigenetic chemical modification in the brain (Hahn *et al*. 2013). 5hmC is generated by ten‐eleven translocation protein (TET)‐dependent 5mC oxidation. The TET family includes the three dioxygenases TET1–3 that convert 5mC to 5hmC in a Fe(II)‐ and α‐ketoglutarate‐dependent manner (Tahiliani *et al*. 2009). In the mammalian brain 5hmC accounts for 1% of all cytosines in cortical DNA (which is equal to ~ 20–25% of total 5mC) and the relative levels of 5mC versus 5hmC are implicated in the regulation of cortical neurogenesis (Jin *et al*. 2011). TET2 and 3 are highly expressed during cortical neurogenesis, with increasing expression levels from progenitors to neurons. TET2 is most prevalent in outer cortical layers, whereas *Tet3* is broadly expressed in all cortical layers (Hahn *et al*. 2013; Diotel *et al*. 2017). Accordingly, RGPs in the VZ and young neurons in the intermediate zone contain lower concentration of 5hmC as compared to neurons in the CP. Intragenic 5hmC‐enriched genes are associated with higher transcript levels than others and include many genes critical for neuronal differentiation, migration or axon guidance. Recent evidence suggests that increased TET activity and reduced levels of Polycomb‐mediated repressive histone methylation (discussed in more detail below) work in a synergistic manner to promote neuronal differentiation (Hahn *et al*. 2013). How TETs regulate lineage priming and qualitative RGP output remains an important unsolved question and requires the analysis of loss and gain‐of TET function at single cell resolution.

5hmC is oxidized to generate 5‐formylcytosine (5fC) and 5‐carboxylcytosine (5caC). Both 5fC and 5caC are recognized and excised by thymine DNA glycosylase (TDG). TDG coupled with base excision repair substitutes 5fC and 5caC by an unmodified cytosine, resulting in DNA demethylation (He *et al*. 2011; Nabel *et al*. 2012). During embryonic development, 5hmC and 5caC levels are inversely correlated in different cell types. While RGPs are almost completely devoid of 5caC, this mark accumulates during lineage specification at cell type specific promotors. Accumulation of 5caC at promoters of key glial markers correlates with high transcript levels and glial differentiation. However, it remains unclear whether increased 5caC is a cause or consequence of glial differentiation, or whether a third mechanism could drive both potential responses independently. Experimental evidence from genetic studies suggests that 5caC drives RGP lineage progression towards gliogenesis, since TDG knock‐down results in 5caC retention and enhanced glial differentiation (Wheldon *et al*. 2014). Future studies will be required to mechanistically dissect the causal link between 5caC levels and astroglial production in more detail.

## Role of genomic imprinting in neural stem cell proliferation behavior

Besides global effects on gene expression, differential DNA methylation at imprinting control regions serves as fundamental regulator of genomic imprinting. Imprinting results in parent‐of‐origin specific gene expression where certain genes are expressed solely from the paternally inherited allele and others only from the maternally inherited allele (Barlow and Bartolomei 2014). A key characteristic of imprinted genes is reflected in their cardinal gene‐dosage sensitivity. A number of imprinted genes have been shown to play critical roles in neurogenesis and neuronal differentiation including cyclin‐dependent kinase inhibitor 1c (*Cdkn1c*), zinc finger protein regulating apoptosis and cell cycle arrest (*Zac1*), delta‐like homologue 1 (*Dlk1*) and insulin‐like growth factor 2 (*Igf2*).

The *Cdkn1c* gene (aka *p57* ^*KIP2*^) is a member of the CDK interacting protein/kinase inhibitory protein (CIP/KIP) family of cyclin‐dependent kinase inhibitors which regulate G1/S transition by inhibiting cyclin/CDK complexes (Sherr and Roberts 1999). *Cdkn1c* is maternally expressed in the developing cortex from E11.5 onwards, with highest expression at E14.5 in RGP and IP nuclei. *Cdkn1c* ^*−/−*^ mice exhibit macrocephaly with disrupted cortical lamination resulting from increased RGP proliferation because of decreased overall cell cycle length and shortening of G1 phase (Mairet‐Coello *et al*. 2012). *Cdkn1c* has been shown recently to mark slowly dividing prospective post‐natal precursors which emerge from progenitors located in the ganglionic eminence (Furutachi *et al*. 2015). Despite the fact that such slowly dividing stem cell precursors have been identified in the developing cortical VZ (Fuentealba *et al*. 2015) it is not clear whether and how *Cdkn1c* instructs cortical RGP lineage progression.

The gene encoding *Zac1* is expressed from the paternal allele with particular high expression in neuroectodermal stem cells during early development (Valente *et al*. 2005). Full knockout of *Zac1* results in hydrocephaly and decreased brain size, whereas *Zac1* overexpression in RGPs triggers premature cell cycle exit because of induction of *Cdkn1c* expression (Daniel *et al*. 2015; Rraklli *et al*. 2016). It is an intriguing hypothetical concept that the expression level of one imprinted gene (*Zac1*) regulates the expression of a second dosage‐sensitive imprinted gene (*Cdkn1c*) to modulate unitary RGP output. Furthermore, independent of *Cdkn1c, Zac1* negatively controls the neurogenic to astrogenic switch in proliferating RGPs by inducing expression of the JAK/STAT3 signaling inhibitor *Socs3* (Schmidt‐Edelkraut *et al*. 2013).

*Dlk1* encodes a transmembrane protein of the Notch/Delta/Serine signaling family. Two different isoforms, a membrane‐bound form and a secreted form have been identified (Smas *et al*. 1997; Wang and Sul 2006). *Dlk1* exhibits paternal specific expression throughout embryonic development (Kobayashi *et al*. 2000) but allele specific expression of *Dlk1* is lost in adult NSCs. Biallelic *Dlk1* expression is required for post‐natal SVZ neurogenesis and OB neuron production (Ferrón *et al*. 2011). However the underlying mechanisms how *Dlk1* gene dosage controls stem cell proliferation behavior remain to be determined.

*Igf2* encodes a potent growth factor promoting cell survival, proliferation, and differentiation upon binding to insulin‐like growth factor receptors (Nielsen 1992; Daniel *et al*. 2015). IGF2 binding to IGF1R positively stimulates growth signaling whereas IGF2 binding to IGF2R results in internalization and lysosomal degradation of IGF2, thereby reducing the growth signal (Stewart and Rotwein 1996). In the embryonic brain, *Igf2* is expressed from the paternal allele but exhibits biallelic expression shortly after birth and switches to maternal expression in the post‐natal brain (Andergassen *et al*. 2017). During corticogenesis, IGF2 is secreted from the choroid plexus into the ventricular CSF thereby stimulating the proliferation of RGPs via IGF1R. *Igf2* ^*−/−*^ mice display reduced brain size, decreased numbers of dividing progenitors and diminished numbers of upper‐layer neurons (Lehtinen *et al*. 2011). In future studies it will be important to decipher the precise functional role of *Igf2* gene dosage in controlling embryonic RGP proliferation behavior and the generation of the correct number of distinct classes of upper‐layer neurons. Interestingly, during post‐natal neurogenesis, biallelic *Igf2* expression is required for adult NSC proliferation (Ferron *et al*. 2015).

In summary, specific imprinted genes have been shown to regulate RGP and adult NSC proliferation behavior and thus their quantitative and qualitative output. The above cited work also supports the hypothesis that imprinted genes encoding for signaling molecules require biallelic expression in adult NSCs to maintain proper OB neuron generation. The control of imprinted gene expression dosage through epigenetic DNA modification represents an intriguing regulatory module with high potential to regulate the generation of cell‐type diversity during cortical development.

## DNA topology controlling RGP lineage progression

The 4D DNA topology orchestrates the ultimate structure and organization of chromatin. Certain genomic regions contact each other in so‐called topologically associated domains (TADs). Cohesin and CCCTC‐binding factor (CTCF) are required for TAD formation and enhancer‐promoter interactions. Association of CTCF to its consensus sequence (three regularly spaced CCCTC repeats) induces cohesin recruitment and formation of a ring‐like structure around distinct sites of the chromosome, thereby inducing DNA looping (Ong and Corces 2014). TAD formation and DNA looping are regulated via modulation of accessibility of CTCF association sites. DNA methylation at CTCF binding sites prevents the interaction of CTCF with DNA (Bell and Felsenfeld 2000; Wang *et al*. 2012), thus excluding the formation of TAD boundaries at methylated DNA sequences. > 77 000 CTCF binding sites widely distributed throughout the genome have been mapped so far (Chen *et al*. 2012; Wang *et al*. 2012). CTCF is highly expressed during neocortical development (Sams *et al*. 2016) and modulates RGP output by maintaining the progenitor state (Watson *et al*. 2014). Qualitatively, CTCF instructs cortical cell‐type diversity by promoting fate specification of post‐mitotic neurons through regulation of genes involved in cell adhesion, 58% of those being c*Pcdh* genes. Almost all promoters of stochastically expressed c*Pcdh* isoforms contain a CTCF‐binding site and *Ctcf* deletion in post‐mitotic neurons leads to misexpression of c*Pcdh* genes and concomitant absence of barrel structures despite layer IV presence (Hirayama *et al*. 2012). Intriguingly, *Dnmt3b* deletion also leads to altered c*Pcdh* expression (Toyoda *et al*. 2014), providing evidence for a potential functional link between DNMT3B and the accessibility of CTCF binding sites which in turn may control c*Pcdh* expression.

## The role of histone modifications in RGP proliferation behavior

N‐terminal histone tails are targets for a variety of post‐translational modifications including the reversible covalent attachment of methyl‐, acetyl‐, phospho‐ or ubiquitin groups to distinct lysine (K) or arginine (R) residues (Yao *et al*. 2016). Such histone modifications activate or repress gene expression (Kouzarides 2007; Karlić *et al*. 2010; Portela and Esteller 2010).

Methylation of histones is catalyzed by histone methyltransferases and reversed by histone demethylases. The most extensively studied histone methylation sites include histone H3 lysine 4 (H3K4), H3K9, H3K27, H3K36, H3K79 and H4K20. Methylation of each of these distinct lysine residues influences the accessibility of chromatin in a different manner. Generally, H3K4me serves as an active mark, whereas H3K9me2/me3 and H3K27me3 are associated with transcriptional repression (Hyun *et al*. 2017).

#### Repressive histone marks

H3K27me3 is catalyzed by the multisubunit Polycomb repressive complex (PRC)2, which consists of three core subunits: enhancer of zeste 2 (EZH2) or its homolog EZH1, embryonic ectoderm development, and suppressor of zeste 12 (Di Croce and Helin 2013). Both, EZH2 and EZH1, contain a conserved SET domain catalyzing the mono‐, di‐, and tri‐methylation of H3K27. PRC1 binds to H3K27me3 and catalyzes the mono‐ubiquitinylation of lysine 119 of histone H2A (H2AK119ub) through a homolog of Drosophila RING protein, thereby ultimately inducing transcriptional silencing. Methylation of H3K27 is reversible, with the two proteins JMJD3 and UTX acting as H3K27 demethylases (Di Croce and Helin 2013).

*Ezh2* and *Ring1B* show high expression in RGPs up to E14.5 and have been proposed to regulate RGP identity and proliferation behavior, as well as RGP‐to‐glial‐progenitor transition (Hirabayashi *et al*. 2009; Pereira *et al*. 2010). Ablation of *Ezh2* and thus H3K27me3 in RGPs correlates with premature RGP differentiation, increased generation of lower‐layer neurons, decreased upper‐layer neuron production, and precocious astrocyte generation (Pereira *et al*. 2010; Hahn *et al*. 2013). *Ezh2*‐mediated repression of gene expression in cortical RGPs is therefore essential for controlling lineage progression and appropriate neuron and glia output. In a complementary experiment with specific deletion of the SET domain of *Ezh2* during mid neurogenesis, RGPs fail to downregulate proneurogenic *Ngn1* signaling, which leads to the suppression of glial cell generation (Hirabayashi *et al*. 2009). Deletion of *Ring1B* during mid neurogenesis does not alter RGP maintenance, but results in alterations of timed production of specific projection neuron populations such as sustained production of CTIP2^+^ layer V neurons (Morimoto‐Suzki *et al*. 2014) and BRN2^+^ upper‐layer neurons (Hirabayashi *et al*. 2009). Similar to deletion of the *Ezh2* SET domain, *Ring1B*‐deficient cortices display defective RGP lineage progression from the neurogenic to the gliogenic state, presumably by failing to suppress proneurogenic genes (Hirabayashi *et al*. 2009). The precise mechanisms by which PRC instructs RGP proliferation behavior are unknown. It is however an attractive hypothesis that PRC association with target genes is differentially regulated in progenitors at distinct stages and post‐mitotic cells, respectively. Key questions that require in‐depth analysis in the future are: (i) How do PRC complexes recognize their target genes? (ii) Which co‐factors regulate PRC recruitment? (iii) How is PRC activity modulated at distinct neurogenic and gliogenic stages? A functionally relevant group of PRC co‐factors in RGPs are chromodomain‐helicase‐DNA‐binding proteins (CHDs) that will be discussed in more detail below.

Repressive H3K9me2/me3 marks are established by the methyltransferases SETDB1, SUV39H1, G9a and G9a‐like protein. H3K9me3 binds heterochromatin protein 1 for transcriptional repression leading to formation and maintenance of heterochromatin. Similar to H3K27me, H3K9me is a reversible mark (Hyun *et al*. 2017). *Setdb1* is highly expressed in proliferating NESCs in the VZ at E9.5 but its expression declines at E15.5 and is not detectable at E17.5. While deletion of *Setdb1* does not affect RGP numbers, it leads to increased upper‐layer neuron production at the expense of deep‐layer neurons. Furthermore, ablation of *Setdb1* causes accelerated astrogliogenesis, demonstrating that *Setdb1* not only regulates the timing of late neurogenic events, but also neurogenic RGP‐to‐astrogenic‐progenitor transition (Tan *et al*. 2012). At the molecular level, SETDB1 catalyzes H3K9 methylation at promoters of glial differentiation genes (e.g. *Sox9* and *Gfap)*, resulting in their repression during neurogenic stages. Taken together, repressive H3K27me3 and H3K9me3 marks correlate with inhibition of precocious neuronal differentiation and controlled timing of gliogenesis. Future studies should aim at identifying functionally‐relevant SETDB1 targets and how these regulate RGP proliferation behavior and lineage progression.

#### Activating histone marks

Transcriptionally active loci are associated with acetylation of histone lysines, e.g. H3K27ac, mediated by histone acetyl transferases and reversed by histone deacetylases (HDACs) (Wang *et al*. 2009). Both types of enzymes are recruited to their target promoters through interaction with sequence‐specific transcription factors.

The gene encoding the histone acetylase cAMP‐response element binding protein binding protein (*Cbp*) is expressed in proliferating RGPs and post‐mitotic neurons during corticogenesis and induces acetylation of H3K9, H3K14 and H3K27 within target gene promoters, such as α*1‐tubulin* (acetylation peak at E13‐E16), *Gfap* (peaking at E16‐P3) and *Mbp* (peaking at post‐natal stages). *Cbp* knockdown or haploinsufficiency diminishes the acetylation levels at those promoters and concurrently leads to reduced production of late‐born upper‐layer neurons from RGPs, as well as decreased transition to glial progenitors (Wang *et al*. 2010). How CBP targeting specificity is achieved by temporally controlled expression of binding partners represents an important line of future research. A key candidate in this regard is NGN1 which prevents interaction of CBP with STAT proteins and subsequent activation of astrogenic gene expression (Sun *et al*. 2001).

## Chromatin remodeling complexes controlling RGP lineage progression

Chromatin remodeling is mediated by multi‐subunit protein complexes including the nucleosome remodeling deacetylase (NuRD) and the Brahma‐associated factors (BAF) complex. The NuRD complex consists of lysine‐specific histone demethylase 1A (LSD1), HDAC1/2, the histone binding proteins RBAP46 and 48, metastasis‐associated protein, methyl‐CpG‐binding domain protein 3, and a CHD protein (Lai and Wade 2011). The BAF complex consists of BRG1, BRM and several distinct BAF proteins (Kadoch *et al*. 2013). Both complexes exhibit alternative subunit composition with temporally regulated expression during development (Son and Crabtree 2014; Nitarska *et al*. 2016).

### NuRD complex

The demethylase LSD1 is expressed in RGPs and post‐mitotic neurons populating the developing CP. LSD1 specifically removes activating H3K4me2 marks from promoters of either neurogenic differentiation‐inducing genes (Zhang *et al*. 2014) or progenitor‐maintaining genes (Wang *et al*. 2016). Targeting of specific promoters by LSD1 is mediated by co‐factors, such as REST corepressor (RCoR)2 (Qureshi *et al*. 2010). The promoters of several RCoR2 target genes including dorso‐ventral CNS specification genes such as *Dlx2, Dlx5, Shh* and *Ascl1*, are transcriptionally repressed by removal of activating H3K4me marks through LSD1. Thus genes maintaining RGP stem cell state, e.g. Sonic Hedgehog (SHH) pathway components are upregulated, whereas genes positively involved in neurogenesis, such *as Emx1, Tbr2, Trnp1, Foxg1* and *Reln*, are downregulated in *Rcor2* knock‐out mice (Wang *et al*. 2016).

HDAC1 and 2 are both expressed in RGPs throughout embryonic development. Together LSD1 and HDACs interact with RCoR1/2 (Qureshi *et al*. 2010). Conditional *HDAC1/2* double knockout mice recapitulate the phenotype observed in *RCoR1/2* double knockouts, characterized by microcephaly caused by a massive block of projection neuron and oligodendrocyte production (Monaghan *et al*. 2017), severe laminar disorganization, and accompanied by a global increase in histone acetylation marks (Montgomery *et al*. 2009; Hagelkruys *et al*. 2014). HDAC1/2 appears to regulate lineage priming by orchestrating neurogenesis at the level of both, RGP cell fate maintenance and specification of distinct neuronal subtypes. As such, removal of histone acetylation promotes layer II/III callosal projection neuron development by inhibiting subcerebral projection neuron fate specification. HDACs are recruited by LHX2, SATB2 and SKI to mediate NuRD complex‐dependent silencing of *Fezf2, Ctip2* and *Sox11* subcerebral projection neuron specification genes (Alcamo *et al*. 2008; Britanova *et al*. 2008; Baranek *et al*. 2012; Muralidharan *et al*. 2017). In OPCs HDACs compete with β‐catenin for TCF7L2 interaction. While the β‐catenin‐TCF complex activates the negative oligodendrocyte differentiation regulator *Id2*, TCF‐HDAC suppresses *Id2* transcription and thus allows oligodendrocyte production (Ye *et al*. 2009). In summary, the interaction of LSD1, HDAC1/2 and RCoR1/2 activates critical temporal gene expression programs that may impact on lineage priming and lineage instruction in RGPs.

The CHD family is characterized by tandem chromodomains and a SNF2‐like ATPase domain (Murawska and Brehm 2011). CHDs exhibit subunit‐specific functions and display mutually exclusive occupancy within the NuRD complex at different stages of corticogenesis (Nitarska *et al*. 2016). Thus CHD proteins have been implicated in modulating the overall output of proliferating RGPs. Indeed, based on loss of function studies, CHD2 and CHD7 have been proposed to regulate self‐amplification of RGPs and prevent precocious cell cycle exit (Micucci *et al*. 2013; Shen *et al*. 2015; Ohta *et al*. 2016). In contrast, CHD3 controls the timing of upper‐layer neuron specification (Nitarska *et al*. 2016) and CHD4 maintains neurogenic RGP fate in an *Ezh2*‐dependent fashion (Sparmann *et al*. 2013). These findings indicate that CHDs interact with PRC and regulate H3K27me3 deposition at target promoters, a hypothesis further supported by recent studies on CHD5 and CHD8. *Chd5* is expressed in neurons throughout cortical development and promotes SATB2^+^ upper‐layer projection neuron production. CHD5 is required to activate expression of genes essential in neuron production, migration and differentiation (such as *Tubb3*,* NeuN* and *Ncam)*, but at the same time to also induce PRC‐mediated silencing of a small cohort of genes involved in development of non‐neuronal lineages (Egan *et al*. 2013). *Chd8* is strongly expressed around the transition from symmetric proliferative to asymmetric neurogenic RGP division (Sugathan *et al*. 2014) and promotes the expression of PRC2 components EZH2 and suppressor of zeste 12. Similar to *Ezh2* deletion (Pereira *et al*. 2010; Hahn *et al*. 2013), knockdown of *Chd8* results in premature depletion of RGPs and impaired neurogenesis (Durak *et al*. 2016). In contrast, twofold reduction of CHD8 protein by *Chd8* haploinsufficiency (deletion of exon 5) or *Chd8* heterozygous loss‐of‐function mutations in humans causes macrocephaly by increasing proliferation of neural progenitors (Katayama *et al*. 2016; Gompers *et al*. 2017; Platt *et al*. 2017). At first glance the results obtained by the knockdown and haploinsufficiency studies appear contradictory. However one may hypothesize that differential gene dosage of CHD8 results in distinct RGP proliferation dynamics. Whereas substantial depletion of CHD8 drastically impairs RGP lineage progression, twofold protein reduction might just delay activation of neuronal differentiation programs. Thus, determining the precise function of CHD8 in controlling RGP proliferation behavior and unitary neuron output remains an important task for further studies.

### BAF complex

In the developing neocortex, distinct BAF subunits are expressed in a temporal and cell‐type specific manner. Proliferating RGPs and post‐mitotic neurons contain BAF complexes with distinct subunit composition, with the RGP BAF complex containing BAF45a and BAF53a, and neuron BAF complex including BAF45b, BAF45c and BAF53b (Lessard *et al*. 2007). BAF45a promotes progenitor cell proliferation and transition from neurogenic to gliogenic RGP cell fate in a BRG‐dependent manner. Knockdown of progenitor BAF components in RGPs results in slow‐down of the cell cycle and overall decrease of proliferating RGPs, thereby strongly reducing the numbers of IPs and upper‐layer neurons (Lessard *et al*. 2007; Matsumoto *et al*. 2016). *Brg1*‐deficiency in embryonic RGPs inhibits the neurogenic to gliogenic switch but E16.5 cortical cultures lacking *Brg1* are not impaired in astrocyte generation (Lessard *et al*. 2007). These findings suggest that niche‐derived signals determine the fate of *Brg1*‐deficient RGPs *in vivo*. Progenitor BAF complex controls RGP proliferation and maintenance on different mechanistic levels: (i) by activating transcription of stem cell differentiation inhibitor *Mash1* (Matsumoto *et al*. 2006, 2016); (ii) by stimulating expression of Notch‐dependent proliferation‐promoting signals and (iii) by repressing SHH‐dependent differentiation‐promoting signals (Lessard *et al*. 2007). During RGP lineage progression, BRG1 also controls OPC specification and oligodendrocyte formation by suppressing precocious *Olig2* transcription (Matsumoto *et al*. 2016). Intriguingly, progenitor BAF displays mutually exclusive incorporation of either BAF170 or BAF155 at distinct developmental stages. Conditional *BAF155*/*170* double mutants display reduced numbers of proliferative RGPs, dramatic thinning of the cortical SVZ and extensive loss of projection neurons, emphasizing a crucial function of BAF complexes in corticogenesis (Narayanan *et al*. 2015). Conditional *BAF155/170* deletion is accompanied by a global shift from activating H3K9ac to repressive H3K27me2/me3 marks (Nguyen *et al*. 2016). What are the exclusive functions of BAF170 and BAF155, respectively, during RGP‐mediated neurogenesis? Neural progenitor BAF complexes harbor BAF170 until E14.5 to repress IP generation by inhibiting the expression of many genes typically activated by PAX6 during upper‐layer neuron development (e.g. *Tbr2*,* Cux1* and *Tle1*) in a BRM‐dependent manner. BAF170 and PAX6 recruit the REST repressor complex to the promoters of target genes, which induces transcriptional silencing of genes involved in late neurogenic events. Between E14.5 and E15.5, BAF170 is replaced by BAF155, which activates expression of IP‐inducing PAX6 target genes in RGPs via association with the H3K27 demethylases JMJD3 and UTX (Lee *et al*. 2007; Tuoc *et al*. 2013; Narayanan *et al*. 2015). Taken together, expression of distinct BAF subunits correlates with the timed generation of cortical projection neuron subtypes; and the interaction of PAX6 with progenitor BAF complexes plays a role in the maintenance of the neurogenic fate of adult NSC‐derived neuroblasts. Upon deletion of either *Pax6* or *Brg1* from adult NSCs, neuroblasts located outside of the neurogenic niche differentiate to glial lineages, especially OPCs (Ninkovic *et al*. 2013). Progenitor BAF complexes thus generally regulate cell‐type diversity by promoting neurogenic fate.

## Control of RGP proliferation behavior by long non‐coding RNAs

Long non‐coding RNAs (lncRNAs) are untranslated transcripts longer than 200 nucleotides modulating chromatin organization, gene transcription, pre‐mRNA metabolism, and RNA turnover (Grammatikakis and Gorospe 2016). The mammalian genome encodes for thousands of lncRNA, most of which are expressed in the brain (Aprea and Calegari 2015). Recent RNA‐seq experiments using human samples at distinct developmental stages revealed that only a few lncRNAs are abundantly expressed in all cortical cell types (e.g. *Norad* and *Brn1b*), whereas the majority of lncRNAs display highly cell type specific expression (e.g. *Pnky* and *LOC646329* in RGPs) (Liu *et al*. 2016). Several lncRNAs have been shown to play important regulatory functions in cortical development *in vivo*. For instance the nuclear lncRNA *Pinky* (*Pnky*) has been implicated in the promotion of RGP stem cell maintenance, presumably by interacting with the RNA splicing factor PTBP1 albeit the precise mechanism how *Pnky* controls RGP proliferation behavior remains to be elucidated (Ramos *et al*. 2015). The long intergenic ncRNA (lincRNA) *Brn1b* (aka *Dali* in humans) is expressed in the developing brain from E13.5 until E18.5 and modulates RGP turnover by promoting expression of the neighboring *Brn1* gene. Deletion of linc‐*Brn1b* suppresses IP generation, leading to abnormal cortical lamination particularly affecting upper‐layer neurons and barrel cortex organization (Sauvageau *et al*. 2013). Since the corresponding human gene product *Dali* interacts with DNMTs (Chalei *et al*. 2014), an appealing hypothesis may propose that linc‐*Brn1b* regulates barrel cortex structures through DNA‐methylation dependent *cPcdh* expression in upper‐layer neurons. Similar to linc‐*Brn1b* and *Brn1b*, many lncRNAs share identical expression patterns with specific neurogenic genes, suggesting that distinct lncRNAs exert a general regulatory function in cell fate (Aprea *et al*. 2013; Liu *et al*. 2016). The cytoplasmic non‐coding RNA activated by DNA damage (*Norad*) is highly expressed in neuronal tissues (Tichon *et al*. 2016) and antagonizes the activity of the RNA binding proteins Pumilio 2 and 3, which are negative regulators of mRNA translation (Lee *et al*. 2016). Cortical RGPs are transcriptionally primed to generate diverse types of neurons by simultaneously expressing mRNA of transcriptional regulators of both deep and superficial layer neurons. As such, the Pum2/E4‐T complex promotes translational repression of deep‐layer fate in upper‐layer neurons, thereby controlling correct temporal specification of newborn upper‐layer neurons (Zahr *et al*. 2018). It will be important in future studies to further elaborate whether or how *Norad* contributes to Pum2 target recognition and which exact role *Norad* exerts in generating cortical cell‐type diversity.

## Conclusions and perspectives

**Figure 2:**
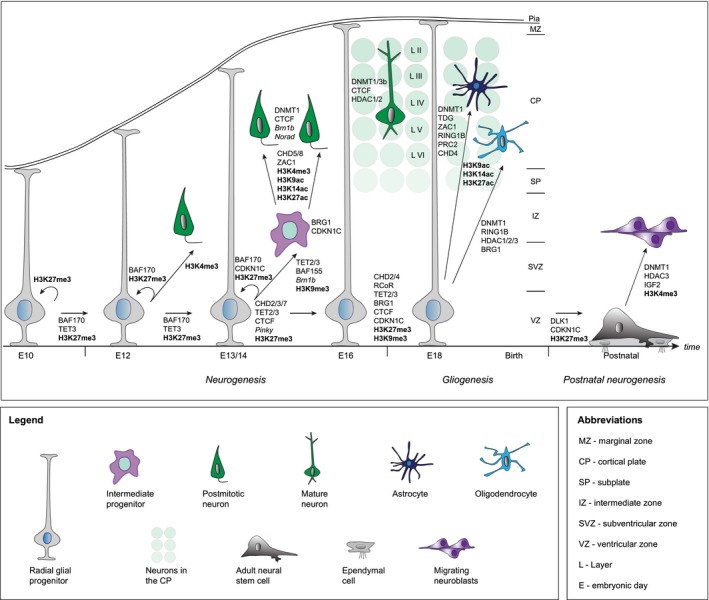
Epigenetic factors and modifications regulating the generation of cell‐type diversity during RGP lineage progression. High content of repressive H3K9me2/me3 and H3K27me3 marks and the presence of chromatin regulators such as BAF170, BRG1, CHD2, *Pnky*, TET1/3 and CTCF regulate stem cell maintenance and RGP self‐renewal while suppressing genes involved in neuron differentiation. For neuron production, repressive marks are replaced by active marks such as H3K4me3 or histone acetylation to promote expression of proneural genes mediating neuronal differentiation and maturation. Transition from repressive to activating epigenetic regulation is mediated through BAF155, BRN1, CHD5/8 and CBP. PRC and histone acetylation are essential for mediating the neurogenic to gliogenic transition. Adult NSCs display high levels of H3K27me3 and require the accumulation of H3K4me3 and expression of DNMT1, HDAC3, and CDKN1C for the faithful generation of OB inhibitory neurons. BAF, Brahma‐associated factor; Cdkn1c, cyclin‐dependent kinase inhibitor 1c; CHD, chromodomain‐helicase‐DNA‐binding; CTCF, CCCCTC‐binding factor; DNMT, DNA methyltransferase; HDAC, histone deacetylase, NSCs, neural stem cell; PRC, polycomb repressive complex; RGP, radial glial progenitor; TET, ten‐eleven translocation protein.

The mammalian cerebral cortex consists of an extraordinary diversity of neurons and glial cells. However, the complete picture of cortical cell‐type diversity is just emerging. While cortical laminar position enables a rough classification, many other criteria ranging from morphological and physiological to transcriptomic and epigenetic fingerprinting have also been employed. In particular, single cell RNA sequencing has greatly transformed our understanding of cell‐type diversity in the developing and adult cerebral cortex (Poulin *et al*. 2016; Lein *et al*. 2017; Zeng and Sanes 2017). While single cell transcriptomes and methylomes (Luo *et al*. 2017) represent a robust measure to classify cell types, the mechanistic principles controlling their generation by RGPs *in vivo* remain mostly unclear. In Fig. 2, we summarize the most important epigenetic modulators and their proposed function in distinct steps of RGP lineage progression. RGPs display silencing of genes mediating post‐mitotic cell fates by maintaining repressive DNA methylation, H3K9me, and H3K27me marks. Successive temporally controlled production of neuronal and glial subtypes requires selective removal of those repressive modifications and the addition of activating H3K4me or acetylation marks at specific target loci. At the same time, genes conferring alternative cell fates need to be silenced. Target specificity can be mediated by expression of mutually exclusive subunits of large epigenetic complexes or by distinct co‐factors serving as recruitment hubs for specific epigenetic modulators.

Recent single cell lineage tracing approaches (Woodworth *et al*. 2017) including MADM‐based experimental paradigms (Hippenmeyer *et al*. 2010, 2013; Gao *et al*. 2014; Beattie *et al*. 2017) have revealed a rough inaugural quantitative framework of RGP lineage progression. Over the last years, it became apparent from genetic studies that RGP lineage progression is modulated by epigenetic components. We propose three major hypothetical conceptual frameworks how epigenetic cues may control RGP‐mediated generation of cortical cell‐type diversity: (i) direct but progressive distinct RGP‐mediated lineage instruction at the time of neuron/glia production; (ii) epigenetic pre‐priming of RGPs which functionally only precipitates at a later stage in the lineage and (iii) priming of an entire successive RGP lineage at a defined developmental stage (Fig. 1). Many past studies focused on the analysis of global knockdown or genetic loss of function of epigenetic regulators and therefore little is known about the functional epigenetic mechanisms at the single RGP level. In order to probe the function of genes encoding epigenetic regulators at single cell level *in vivo*, MADM technology may offer a promising approach for future analysis. Despite that epigenetic processes regulate the expression of downstream target genes it is currently not clear how the precise epigenetic state of a proliferating RGP correlates with its neuron/glia output. The epigenetic landscape is highly dynamic and even during distinct phases in the RGP cell cycle crucial transcriptional changes associated with differences in the epigenetic marks may be required for correct lineage progression and/or priming. It remains a substantial challenge to rigorously analyze transcriptome and epigenome fingerprints in real time and at the single cell level to address the following questions in more detail: What are the precise cell‐autonomous mechanisms regulating RGP output and what are the essential non‐autonomous signals elicited by the stem cell niche? Which epigenetically controlled signaling molecules contribute to RGP lineage progression? How do epigenetic cues contribute to the regulatory process to instruct whether RGPs progress into either astrocyte progenitors or OPCs? In light of the emerging evidence that DNA methylation can affect the modification states on accompanying histones and vice versa (Vaissière *et al*. 2008; Rose and Klose 2014; Nishiyama *et al*. 2016) it will be important to determine whether such interactions play an instructive role in RGP lineage progression and/or post‐mitotic fate specification. The function and impact of distinct histone modifications, in general, on RGP proliferation behavior and beyond requires also more investigation in the future. Furthermore, it will be essential to comprehensively analyze the precise molecular and biochemical function of the various epigenetic protein complexes, described in the above sections, in RGP lineage progression at single cell and high temporal resolution. It will be revealing to more precisely categorize specific epigenetic modulators (Fig. 2) with regard to functional requirement in lineage instruction, lineage pre‐priming or lineage priming (Fig. 1). Lastly, most functional analyses of epigenetic regulators that contributed to our current understanding of RGP lineage progression were reliant on mouse genetic approaches. How are the proliferative RGP potential and the generation of cell‐type diversity regulated in different species? It will be particularly important to analyze epigenetic mechanisms also in higher mammalian brains. Interestingly, neural stem cells in ferret and human were recently shown to express the histone methyltransferase *Prdm16* (Baizabal *et al*. 2018) although the precise role of *Prdm16* in stem cell lineage progression remains somewhat elusive since it was mainly addressed by loss of gene function in mice. Experimental access to the human embryonic brain is extremely limited. Yet, recent advances in pluripotent stem cell technology now enable the generation of cerebral organoids that at least recapitulate some aspects of early‐to mid‐fetal human cortical development (Lancaster and Knoblich 2014; Suzuki and Vanderhaeghen 2015; Quadrato and Arlotta 2017). Therefore, future studies with the goal to contribute to our understanding of the epigenetic mechanisms controlling the generation of cortical neuron and glial cell diversity in distinct species and humans may also help to build a potential foundation for prospective reprogramming and/or stem cell‐based approaches in regenerative medicine.
